# Highly Hydrophilic Gold Nanoparticles as Carrier for Anticancer Copper(I) Complexes: Loading and Release Studies for Biomedical Applications

**DOI:** 10.3390/nano9050772

**Published:** 2019-05-20

**Authors:** Ilaria Fratoddi, Iole Venditti, Chiara Battocchio, Laura Carlini, Simone Amatori, Marina Porchia, Francesco Tisato, Federica Bondino, Elena Magnano, Maura Pellei, Carlo Santini

**Affiliations:** 1 Chemistry Department Sapienza University of Rome, P.le A. Moro 5, 00185 Rome, Italy; ilaria.fratoddi@uniroma1.it (I.F.); simone.amatori@gmail.com (S.A.); 2 Sciences Department Roma Tre University of Rome, via della Vasca navale 79, 00146 Rome Italy; chiara.battocchio@uniroma3.it (C.B.); laura.carlini@uniroma3.it (L.C.); 3 ICMATE, National Research Council (CNR), Corso Stati Uniti, 4-35127 Padua, Italy; francesco.tisato@cnr.it; 4 IOM-CNR Laboratorio TASC, SS 14, km 163,5 Basovizza, I-34149 Trieste, Italy; bondino@iom.cnr.it (F.B.); magnano@iom.cnr.it (E.M.); 5 School of Science and Technology, University of Camerino, 62032 Camerino (MC) Italy; maura.pellei@unicam.it (M.P.); carlo.santini@unicam.it (C.S.)

**Keywords:** gold nanoparticles, copper(I) complexes, conjugates, drug delivery, anticancer compounds

## Abstract

Gold nanoparticles (AuNPs), which are strongly hydrophilic and dimensionally suitable for drug delivery, were used in loading and release studies of two different copper(I)-based antitumor complexes, namely [Cu(PTA)_4_]^+^ [BF_4_]^−^ (A; PTA = 1, 3, 5-triaza-7-phosphadamantane) and [HB(pz)_3_Cu(PCN)] (B; HB(pz)_3_ = tris(pyrazolyl)borate, PCN = tris(cyanoethyl)phosphane). In the homoleptic, water-soluble compound A, the metal is tetrahedrally arranged in a cationic moiety. Compound B is instead a mixed-ligand (scorpionate/phosphane), neutral complex insoluble in water. In this work, the loading procedures and the loading efficiency of A and B complexes on the AuNPs were investigated, with the aim to improve their bioavailability and to obtain a controlled release. The non-covalent interactions of A and B with the AuNPs surface were studied by means of dynamic light scattering (DLS), UV–Vis, FT-IR and high-resolution x-ray photoelectron spectroscopy (HR-XPS) measurements. As a result, the AuNPs-A system proved to be more stable and efficient than the AuNPs-B system. In fact, for AuNPs-A the drug loading reached 90%, whereas for AuNPs-B it reached 65%. For AuNPs-A conjugated systems, a release study in water solution was performed over 4 days, showing a slow release up to 10%.

## 1. Introduction

Gold nanoparticles (AuNPs) are the most versatile material in nanotechnology, with a huge range of biological and biomedical applications, such as diagnostic, therapeutic and biosensing applications [1,2,3,4,5,6,7]. In particular, AuNPs have been often proposed as non-toxic carriers for drug and gene-delivery applications [8,9,10,11,12,13]. In fact, the specific properties of AuNPs, such as their high surface-to-volume ratio, peculiar optical properties, easy synthesis and versatile surface functionalization, hold pledge in the clinical field for cancer therapeutics [14,15]. Moreover, AuNPs present optical properties, which can be easily tuned to desirable wavelengths according to their shape (e.g., nanoparticles, nanoshells, nanorods, etc.), size (e.g., 1 to 100 nm) and composition (e.g., core/shell or alloy noble metals) [16,17,18,19,20], enabling their imaging and photothermal applications [21,22,23,24,25,26]. AuNPs can also be easily functionalized with different moieties, such as antibodies, peptides and/or DNA/RNA to specifically target different cells [10,27,28], and with biocompatible molecules to prolong their in vivo circulation for drug delivery applications [29,30]. Furthermore, it is well known that passive targeting can be achieved by using AuNPs as a carrier, because of their preferential accumulation in tumor cells (enhanced permeability and retention (EPR) effect) [21].

In recent years, the biomedical research of new metal-based anticancer drugs alternative to Pt(II) derivatives (cisplatin, oxaliplatin and carboplatin, which are currently utilized in clinical practice) has been focused on complexes including, among other metals, gold, ruthenium, silver and copper [31,32,33,34,35,36]. The purpose of these studies is to circumvent severe toxicity in non-tumor cells as well as inherited and/or acquired resistance phenomena caused by Pt(II) drugs [37,38,39]. In particular, among the abovementioned metals, copper is receiving increasing attention [36]. Copper, as an essential micronutrient in mammalians, plays a pivotal role in redox-chemistry, growth and development, and is a key co-factor for the function of several enzymes involved in energy metabolism, respiration and DNA synthesis [40]. In addition, homeostatic mechanisms strictly define the concentration of copper in mammalian cells, which have also developed a physiological active transport mechanism for its internalization based on a trans-membrane protein referred to as human copper transporter 1 (hCtr1) [41,42]. Novel copper-based antitumor agents have been studied according to the view that endogenous metals may be less toxic toward normal cells with respect to cancer cells. Since the generally assessed mechanism of copper cell uptake implies the reduction from copper(II) to copper(I) followed by internalization through transmembrane transporters [43,44], our research has been mainly focused on copper(I) derivatives. The synthetic strategy utilizes ligands with soft donor atoms such as phosphorous in tertiary phosphanes or aromatic sp^2^ hybridized nitrogen of pyrazolyl derivatives. Among these compounds, homoleptic, cationic phosphane complexes well match the ability of hCtr1 protein to internalize specifically monovalent ions, thus leading to outstanding cytotoxic efficiency toward cancer cells in both in vitro and in vivo trials [45,46,47,48,49]. In addition, neutral mixed-ligand complexes containing both scorpionate-like (N-donor) and phosphane ligands showed remarkable cytotoxic activity in in vitro and in vivo tests as well [50]. Despite the promising results, open problems remain, such as the low solubility and bioavailability of some of these compounds and their uncontrolled release. In this preliminary work, hydrophilic AuNPs were synthesized and loaded with either a representative of a water-soluble, cationic complex—[Cu(PTA)_4_]^+^ [BF_4_]^−^ (A; PTA = 1,3,5-triaza-7-phosphadamantane)—or a lipophilic, neutral complex—[HB(pz)_3_Cu(PCN)] (B; HB(pz)_3_ = tris(pyrazolyl)borate, PCN = tris(cyanoethyl)phosphane)—aiming at the construction of a novel drug delivery system. The use of hydrophilic AuNPs as a vehicle for copper complexes is an innovative and strategic approach to improve the solubility and stability in water of the copper complexes, and consequently to increase their bioavailability. Moreover, these drug delivery systems allow the investigation of a slow and controlled release of copper complexes, opening the way for promising scenarios of in vivo and in vitro experimentation.

## 2. Materials and Methods

### 2.1. Materials and Characterizations

Sodium 3-mercapto-1-propanesulfonate (HS(CH_2_)_3_SO_3_Na, 3MPS, Aldrich, 99%, St. Louis, MO, USA), tetrachloroauric(III) acid trihydrate (HAuCl_4_·3H_2_O, Aldrich, 99.0%, St. Louis, MO, USA), sodium borohydride (NaBH_4_) and PBS buffer solution at pH = 7.4 (technical grade Aldrich, St. Louis, MO, USA) were used as received. UV–Vis spectra were acquired in H_2_O and MeOH solutions by using quartz cells with a Varian Cary 100 Scan UV–Vis spectrophotometer. The size distribution of AuNPs in H_2_O solution was investigated by means of the dynamic light scattering (DLS) technique by using a Zetasizer Nanoseries Malvern instrument, at the specific temperature (25.0 ± 0.2 °C and 37.0 ± 0.2 °C). Correlation data were acquired and fitted in reference to our previous work [51,52]. Field emission scanning electron microscopy (FESEM) images were acquired with an Auriga Zeiss instrument, resolution 1 nm, applied voltage 6–12 kV) on freshly prepared films drop-cast from a water solution on a metallic sample holder. A Mini Spin-Eppendorf centrifuge was used for the purification of AuNPs samples (13,000 rpm, 20 min, five times with deionized water). Deionized water was obtained from Zeener Power I Scholar-UV (18.2 MΩ). A Scanvac-CoolSafe55-4 Lyophilizer was used to dry samples. Attenuated total reflection (ATR) spectra were recorded with a Bruker Vertex 70 instrument in the range of 4000–400 cm^−1^. High-resolution x-ray photoelectron spectroscopy (HR-XPS) experiments were carried out at the CNR BACH (Beam line for Advanced DiCHroism) [53] undulator beam line at the ELETTRA Synchrotron Radiation facility of Trieste (Italy). XPS data were collected with a pass energy equal to 50 eV, with the monochromator entrance and exit slits fixed at 20 μm. Photon energies of 386 eV, 596 eV and 1022 eV were used respectively for C1s, Au4f, P2p, B1s; O1s, N1s; Cu2p spectral regions, with an energy resolution of 0.23 eV. C1s spectra used for calibration were recorded at all photon energies. Calibration of the energy scale was made by referencing all the spectra to the C1s core level signal of aliphatic C atoms, always found at 285.00 eV, and the Au4f7/2 signal of metallic gold measured on a reference gold foil (84.00 eV Binding Energy, BE) [54,55]. A curve-fitting analysis was performed using Gaussian curves as fitting functions. When several different species were individuated in a spectrum, the same FWHM value was used for all individual photoemission bands. To perform HR-XPS analysis, pristine Cu(I) complexes (A = [Cu(PTA)_4_]^+^ [BF_4_]^−^ and B = [HB(pz)_3_Cu(PCN)] and functionalized Au nanoparticles (AuNPs-A and AuNPs-B) were deposited onto TiO_2_/Si(111) (as to avoid any signal interference) wafer substrates by following a drop-casting procedure.

### 2.2. Preparation of Conjugate Nanoparticles

The AuNPs stabilized with 3MPS were synthesized as previously reported [9]. Briefly, starting with 200 mg (5 × 10^−4^ mol) of HAuCl_4_ × 3H_2_O in 20 mL of deionized water, a solution of 3MPS in 20 mL of deionized water was added under vigorous stirring (Au/S = 1/4 molar ratios). Two hours after the addition of the reduction agent (a water solution of NaBH_4_, Au/NaBH_4_ molar ratio = 1/10), a solid black product was isolated and purified by centrifugation (13,000 rpm, 20 min, five times with deionized water). AuNP main characterizations: UV–Vis (λ_max_ (nm), H_2_O) 523 nm; DLS (<2R_H_> (nm), H_2_O): 12 ± 3 nm; Z potential: –35 ± 3 mV; FESEM (nm) 8–10 nm. Complexes A and B were prepared according to procedures reported in the literature [45,50]. They showed a well-known UV–Vis spectrum, as reported in the Supporting Information, Figures S1a,b). The conjugate nanoparticles were prepared using the following procedure. The AuNPs and A were mixed in water (Au/A = 5/1 w/w) under gentle stirring (room temperature, 4 h) and then the suspension was centrifuged (13,000 rpm, 2 h) to obtain AuNPs-A as a solid residue. It was lyophilized and stored at room temperature, while the supernatant was used for loading evaluations. The AuNPs and B were mixed in MeOH (Au/B= 5/1 w/w) under gentle stirring (room temperature, 4 h) and then the suspension was centrifuged (13,000 rpm, 30 min) to obtain AuNPs-B as a solid residue. It was lyophilized and stored at room temperature, while the supernatant was used for loading evaluations. The loading and the loading efficiency (η) were calculated by using calibration curves, as reported in the Supporting Information (Figures S1c,d) and calculated in reference to our previous work [9]. For each sample, three independent measurements were carried out and the mean value and standard deviation are reported.

### 2.3. Stability and Release Studies

For the stability studies, AuNPs-A and AuNPs-B were dispersed in water at the concentration of 0.1 mg/mL and the size of nanoparticles was measured over 10 days at room temperature. The release studies were performed in H_2_O at 37 °C over 4 days, using 2 mg of conjugated nanoparticles in 20 mL of media. The released was calculated by analyzing the water solution and detecting the free complex by using UV–Vis measurements, in reference to our previous work [9]. For each sample, three independent measurements were carried out and the mean value and standard deviation are reported.

## 3. Results and Discussion

### 3.1. Conjugate Nanoparticles: Preparation, Characterization and Loading Studies

**Figure 1:**
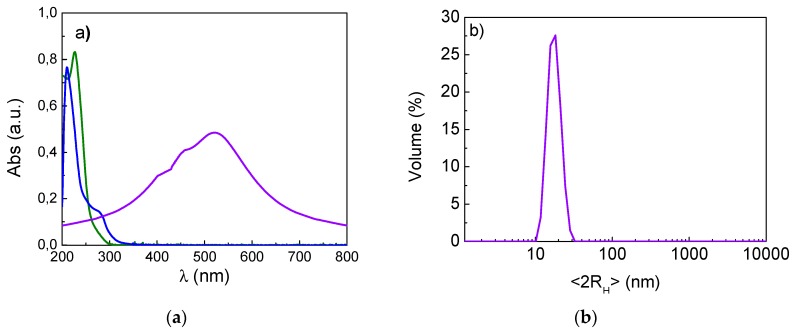
(**a**) UV–Vis spectrum of gold nanoparticles (AuNPs) (violet curve) and complexes A (green curve) and B (blue curve); (**b**) dynamic light scattering (DLS) measurement in water of AuNPs alone (in violet): <2R_H_> = 15 ± 2 nm.

Highly hydrophilic gold nanoparticles were synthetized following a previously published procedure [9], and the UV–Vis spectrum and DLS measurements confirmed their nanodimension, as shown in Figure 1a,b. The results revealed that these AuNPs are particularly suitable for Cu(I) complexes delivery. In fact, AuNPs functionalized by 3MPS showed a high degree of stability and hydrophilicity due to the small length alkyl chains thiol with a charged terminal sulphonate group. Moreover, the 3MPS choice as a ligand, with a molar ratio of Au/S = ¼, guarantees a balance between stability and loading and favours transport in a watery environment. This fact increases the final bioavailability of the conjugates, especially for compounds with low water solubility. Furthermore, the plasmonic absorption peaks of the AuNPs and the absorption peaks of the copper complexes (λ_max_ at 228 nm and 268 nm for complexes A and B, respectively, as shown in Figure 1a and in Figures S1a,b [45,50]) appeared in different areas of the spectrum—in the UV spectrum for complexes and in the visible spectrum for AuNPs, allowing easy detection of the loading processes. This feature makes it possible to design a loading protocol based on the simple physical contact of AuNPs and copper complexes that can be physically adsorbed.

**Figure 2:**
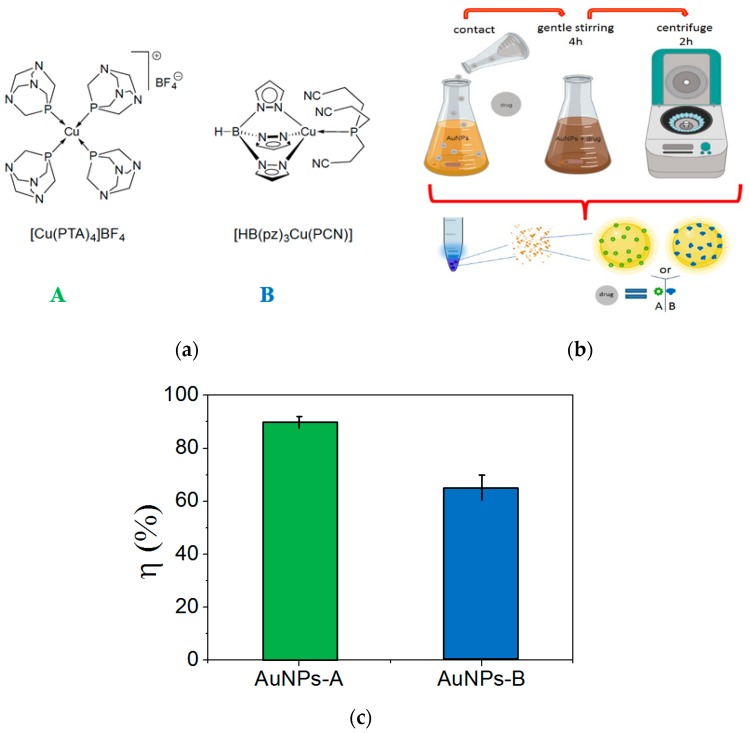
(**a**) Chemical structures of anticancer Cu(I) complexes used in this study; (**b**) sketch of loading protocol to obtain AuNPs-A and AuNPs-B conjugates; (**c**) loading efficiency η (%) for AuNPs-A (in green, η (%) = 90 ± 4 %) and AuNPs-B (in blue, η (%) = 65 ± 10 %).

On the basis of these considerations, the loading protocol for AuNPs and the two Cu(I) complexes was performed in a water solution at room temperature under gentle stirring. In Figure 2a,b, the chemical structures of the anticancer Cu(I) complexes used in this study, A and B, and a sketch of the loading protocol to obtain AuNPs-A and AuNPs-B conjugates are reported. The value of the loading efficiency η (%), reported in Figure 2c, can be calculated as follows [9,13]:

η (%) = (m_loaded drug_/m_drug_) 100,

where m_loaded drug_ is the mass of the loaded drug (A or B), calculated from UV–Vis quantitative data and m_drug_ is the mass of the drug (A or B) used in the experimental procedure. From the absorbance value of free A or B, it is possible to obtain the amount of loaded drug, by determining the difference.

**Figure 3:**
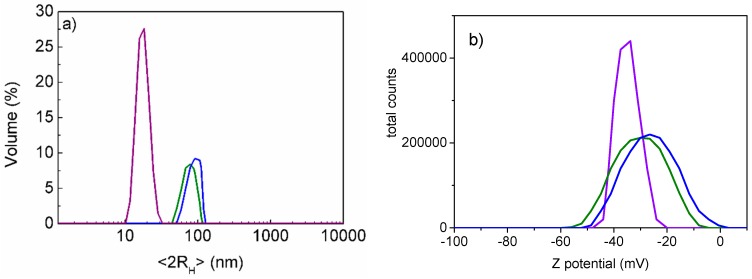
DLS data of AuNPs in violet, AuNPs-A in green and AuNPs-B in blue: (**a**) <2R_H_> in water: AuNPs (15 ± 2 nm), AuNPs-A (56 ± 30 nm) and AuNPs-B (76 ± 32 nm); (**b**) Z potential in water: AuNPs (–35 ± 2 mV), AuNPs-A (–30 ± 3 mV) and AuNPs-B (–23 ± 4 mV).

The loading studies allowed to us obtain the conjugated systems: AuNPs-A with η = 90 ± 4% and AuNPs-B with η (%) = 65 ± 10%. These systems were characterized in depth to understand the chemical interaction between AuNPs and Cu(I) complexes A and B. DLS studies were performed in a water suspension and showed a dimensional increase for AuNPs-A and AuNPs-B compared with the AuNPs alone, as reported in Figure 3a. In fact, the conjugation of the complexes involved a different degree of hydration of the particles and, as a result, the hydrodynamic diameter (<2R_H_>) increased.

Moreover, DLS allowed us to measure the electrophoretic mobility and, using the Smoluchowski equation, the Z potential [51,52]. The Z potential is the potential at this slipping plane, i.e., the surrounding electrical double layer, where the liquid moves together with particles. Therefore, the measured Z potential is not exactly the surface potential (surface charge density), but it is the potential of practical interest because it determines the inter-particle forces and enables the evaluation of the stability of the colloidal system [51,52]. The Z potential studies performed on our conjugates systems confirmed these interactions between AuNPs and complexes A and B. In fact, the Z potential was –30 ± 3 mV and −23 ± 4 mV, respectively, for AuNPs-A and AuNPs-B, instead of −35 ± 2 mV for AuNPs alone, as shown in Figure 3b. This is due to two different effects introduced by the presence of complexes A and B on the surface of the AuNPs. The first effect is the decrease of the negative charge density, due to the presence of neutral or positively charged molecules on the gold surface; this is strictly related to the Stern layer and slipping plane around the nanoparticles, which produce the Z potential. The second effect is the different aggregation grade, also observable from signal enlargement and size measurements, due to the interaction between complex molecules linked on different and vicinal AuNPs, which cause the system to be less stable in general. The balance or the prevalence of one of these two effects also explains the slight difference between the Z potential values of AuNPs-A and AuNPs-B. In fact, the conjugate AuNPs-B showed a larger size and lower Z potential with respect to AuNPs-A (see Figure 3). Indeed, complex A had a positive charge, facilitating adsorption and producing greater loading efficiency. Moreover, complex A on AuNPs decreased the interaction phenomena between the absorbed complex molecules, reducing the aggregation phenomena of the colloidal system in a solution. This justifies the DLS results regarding the smaller dimensions and more negative Z potential of AuNPs-A compared to those of AuNPs-B.

FESEM-EDX investigations performed on conjugate AuNPs-A nanoparticles showed dimensions around 10 nm with the presence of some aggregates (see Figure S2). It can be noticed that the dimensions obtained from DLS were greater than those obtained from FESEM images. Such a dimensional difference is due to the intrinsic difference between the two techniques, based on different principles. In fact, DLS estimated the particles hydrodynamic diameter (<2R_H_>) in the aqueous environment, with the important effect of swelling, and this dimension is the Z average value, which is the mean diameter weighted over the scattered light intensity. On the other hand, microscopy measurements were carried out under a vacuum on a dry sample deposited by casting with no hydration effects. The particles were more or less aggregated due to concentration or to fast or slow solvent evaporation occurring during the preparation of the sample. For this reason, it is difficult to directly compare the two measurements.

The Energy Dispersive X-ray Analysis (EDX) evidenced the presence of Cu and, in particular, the semiquantitative analysis showed the ratio of Au:Cu to be around 0.4:0.03 (see Figure S2).

The ATR data confirmed the effective interaction between copper complexes and AuNPs. In fact, both for AuNPs-A and AuNPs-B, typical bands were found (see Figures S3a,b). Particularly for AuNPs-A, some characteristic signals of the A complex were recognizable, such as the bending of CH_2_ at 1456 and 1418 cm^−1^ and the C–N stretching at 1043 and 1000 cm^−1^, thus confirming the successful conjugation. A shift of the C–N stretching signals was also observed, which moved from 1103 and 947 cm^−1^ in the free complex to 1043 and 1000 cm^−1^ in the conjugate, suggesting a direct involvement of these groups in the interaction with the gold nanoparticle surface. For AuNPs-B, the ATR measurements showed the typical bands at 2480 cm^−1^ due to the B–H stretching, bands at 1502 and 1403 cm^−1^ due to the C–C bond of pyrazole rings and at 1301 cm^−1^ due to C–N stretching. In this case the nitrile stretching showed a shift from 2254 cm^−1^ (free complex) to 2240 cm^−1^ (conjugate system), highlighting the involvement of these groups in the conjugation formation [33].

### 3.2. HR-XPS Studies

Molecular and electronic structure of AuNPs-Cu(I) complexes assemblies were probed by means of synchrotron radiation-induced photoemission spectroscopy (HR-XPS); for comparison, the pristine Cu(I) complexes A and B ([Cu(PTA)_4_]^+^ [BF_4_]^−^ and [HB(pz)_3_Cu(PCN)], respectively) were also investigated. Signals were acquired at C1s, P2p, N1s, B1s, Cu2p and Au4f core levels, and the obtained spectra were analyzed by following a peak fitting procedure that evidenced spectral components arising from atoms in different electronic environments.

C1s and P2p spectra data analysis allowed us to assess the Cu(I) complex stability; as reported in Table S1 in the Supporting Information, P2p and C1s components positions, i.e., BE values, which reflect the molecular composition of the organic ligands, were not affected by the AuNP-Cu(I) complexes interaction. Indeed, the P2p_3/2_ spin-orbit component of the phosphorous signal was always found around 131.50 eV BE (complex A: 131.03 eV; AuNP-A: 131.11 eV; complex B: 131.81 eV; AuNP-B 131.86 eV), as expected from the literature for organic molecules containing P atoms [56]. The observed P2p BE stability allowed us to completely dismiss the occurrence of any degradation effect due to molecule oxidation, which would result in phosphane oxide formation with a noticeable shift in the P2p signal BE towards higher values [56]). P2p spectra are reported in Figure S4 in the Supporting Information. Au4f spectra appeared composed, showing a spin-orbit peak of high intensity due to metallic gold atoms at the nanoparticle cores (Au4f_7/2_ BE = 83.9 eV), and a second signal of low intensity at higher BE values (Au4f_7/2_ BE = 85.1 eV) was associated with partially positively charged gold atoms at the NP surface, as expected from the literature on analogous systems [4]. Copper Cu2p core signals were also acquired; for all samples, a single spin-orbit pair was observed, compatible with Cu(I) ions (Cu2p_3/2_ = 931.5 eV BE) [56]. The Au4f spectrum of AuNP-A and the Cu2p spectra of both AuNP-A and complex A were reported as examples in the Supporting Information (Figure S5).

**Figure 4:**
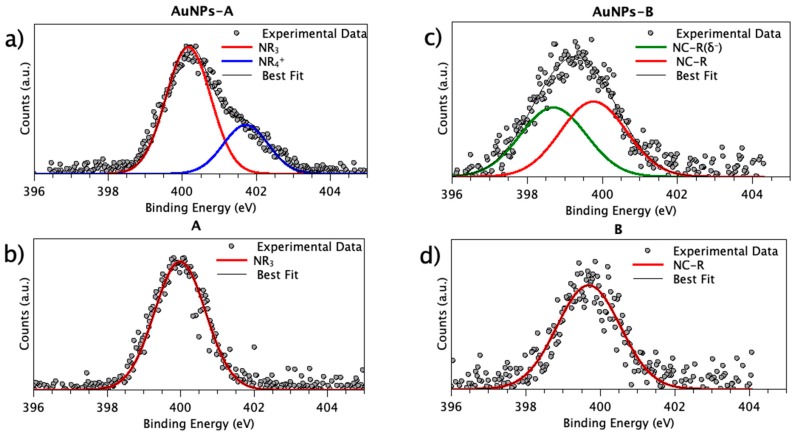
High-resolution x-ray photoelectron spectroscopy (HR-XPS) N1s spectra of: (**a**) AuNPs-A; (**b**) complex A; (**c**) AuNPs-B; (**d**) complex B.

The most interesting signal that shed light into the Cu(I) complex/AuNP interaction was the N1s core level. As reported in Table 1, both Cu(I) compounds showed N1s signals at the BE, as expected in the literature for the proposed molecular structure (tertiary amines N1s are expected at about 400 eV BE, and was found at 399.99 eV for complex A; N ≡ C − R like nitrogen N1s signal is expected at 399.6 eV BE [57], and was found at 399.7 eV BE in complex B [56]). In Figure 4 all N1s spectra are collected. Contributions related to pristine nitrogen atoms in Cu(I) complexes are represented in red.

As evidenced in Table 1 and clearly observable in Figure 4, when Cu(I) complexes (Figure 4b,d) interacted with the gold nanoparticles, the N1s signal shape was modified (Figure 4a,c). For AuNP-A, a shoulder appeared at high BE (Figure 4a), suggesting a new spectral component at about 401 eV BE (in blue in the figure), which is usually assigned to positively charged N atoms in quaternary ammonium salts. On the other hand, the N1s spectrum of AuNP-B was larger at low BE, suggesting that a second spectral component appeared at a lower BE than the pristine N ≡ C − R-like N atom (Figure 4c); this behaviour indicates a partial electron transfer from the AuNPs to the nitrogen atom of the N ≡ C − R functional group.

### 3.3. Conjugate Nanoparticles: Stability and Release Studies

**Figure 5:**
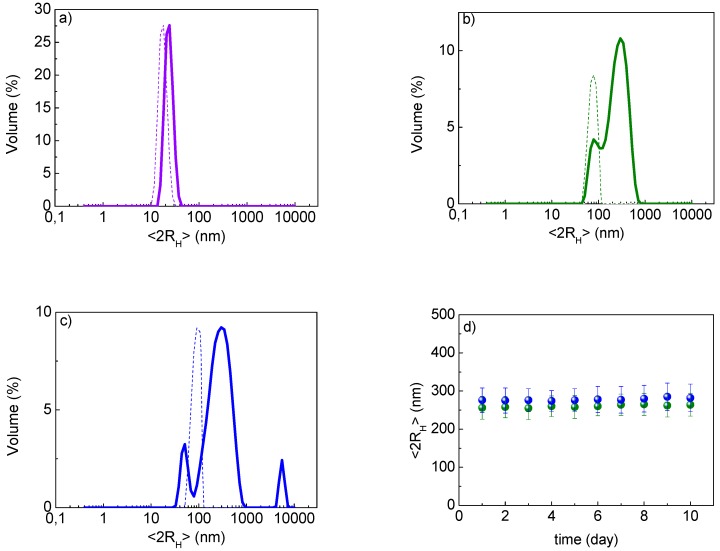
DLS stability study in water of AuNPs in violet, AuNPs-A in green and AuNPs-B in blue: (**a**) AuNPs <2R_H_> before (dashed line) 15 ± 2 nm and after samples lyophilization (solid line) 25 ± 5 nm; (**b**) AuNPs-A <2R_H_> before (dashed line) 56 ± 30 nm and after lyophilization (solid line) 256 ± 30 nm; (**c**) AuNPs-B <2R_H_> before (dashed line) 76 ± 32 nm and after lyophilization (solid line) 276 ± 32 nm; (**d**) <2R_H_> of lyophilized AuNPs-A and AuNPs-B re-suspended in water at different days, up to 10 days.

It must also be highlighted that the main advantage of drug delivery with AuNPs is the possibility of studying a targeted and controlled release in terms of target site and time, as reported in many recent papers [8,9,10]. In this context, it is important to verify the stability of conjugates, also in view of a future formulation that makes them easily storable and at the same time quickly ready for use. The lyophilization appears to fulfil to this requirement, and was therefore performed by studying the effects on AuNPs alone and conjugates, both in terms of dimensional and structural stability. Comparing the fresh samples and the lyophilized samples by DLS measurements, aggregation phenomena were observed with important dimensional variations, as shown in Figure 5a–c. These phenomena involved both AuNPs alone and conjugated systems that remained however with dimensions under 300 nm. Regarding the structural stability of the systems, they showed unaltered structural conformation, confirmed by FTIR measurements, and good results in terms of <2R_H_> reproducibility when they were re-suspended in water at room temperature up to 10 days, as reported in Figure 5d.

**Figure 6:**
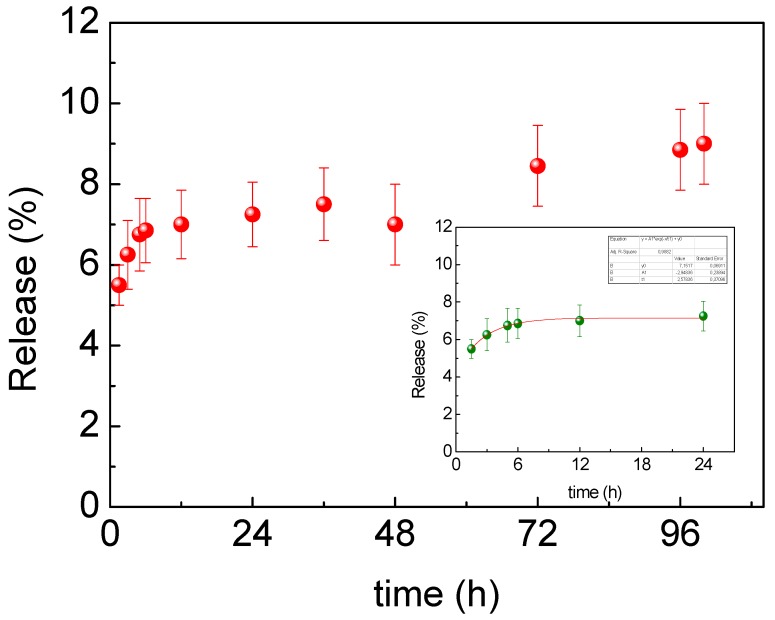
Released profile of AuNPs-A with inset details showing the release in time in the range of 0–24 h.

Therefore, the study on the release was performed. The conjugate was re-suspended in water at 37 °C with gentle stirring and the UV–Vis analysis of the aqueous solution at defined times allowed the quantification of the released complex. This study showed strong and different interactions between Cu(I) complexes A and B and AuNPs, as already evidenced by the HR-XPS studies. In particular, for AuNPs-A, the interaction involved nitrogen that partially transferred electrons to the surface of the metal, creating an interaction that caused a slow release. In Figure 6 the slow release profile of AuNPs-A, less than 10% up to 4 days, is shown. On the other hand, the AuNPs-B system involved the N ≡ C − R moiety, which strongly interacted with the gold surface, making the release not appreciable in a few days (up to 4). Taking these results into account, surely the AuNPs-A conjugate is more promising, not only for the better loading efficiency but, above all, for the evident slow release, unlike the AuNPs-B conjugate.

AuNPs-A showed an excellent and promising result, because with a single administration it could be possible to achieve a slow drug delivery release in a biological site up over 4 days and more. Moreover, a main advantage of delivering a water-soluble drug with AuNPs is the accumulation of AuNPs in cancer cells, which guarantees the drug’s targeting. Further, the slow release is an excellent opportunity to study the synergistic effects of AuNPs and copper complexes, effects that could occur for a long time (days and weeks), as in the case of slow-release anti-inflammatory drugs reported in the literature [9]. This fact opens new scenarios for investigations related to the action mechanisms as well as for synergistic action with AuNPs-A.

## 4. Conclusions

Strongly hydrophilic gold nanoparticles, AuNPs, were prepared to be conjugated with copper(I) complexes. In particular, loading and release studies were performed using two different copper(I) antitumor complexes, namely [Cu(PTA)_4_]^+^ [BF_4_]^−^ (A; PTA = 1,3,5-triaza-7-phosphadamantane) and [HB(pz)_3_Cu(PCN)] (B; HB(pz)_3_ = tris(pyrazolyl)borate, PCN = tris(cyanoethyl)phosphane). In the water-soluble compound A, the metal is tetrahedrally arranged in a cationic moiety, while compound B is a mixed-ligand (scorpionate/phosphane), neutral complex insoluble in water. Loading protocols and efficiency are also related to these structural aspects and were optimized to obtain η = 90 ± 4% and η = 65 ± 10%, respectively, for AuNPs-A and AuNPs-B. Structural differences of A and B induced different behaviours regarding the interactions with the gold surface, as showed by the HR-XPS studies. In fact, for compound A, nitrogen partially transfers electrons to the surface of the metal nanoparticles, creating an interaction that causes a slow release in water, less than 10% in 4 days. On the other hand, in B compound the N ≡ C − R groups hook onto the surface of the gold, producing a strong interaction that makes the release not appreciable in the same time interval (up to 4 days). Therefore, both AuNPs-A and AuNPs-B represent promising examples of water-soluble gold nanocarriers suitable to improve the bioavailability of synthetic drugs, especially considering the EPR effect of AuNPs. In particular AuNPs-A, which achieved a slow release, opens the way for biological in vitro studies to explore the synergic activity of copper complexes and gold nanoparticles.

## Figures and Tables

**Table 1:** N1s BE, FWHM values and assignments for pristine Cu(I) complexes and AuNPs carriers.

Sample	BE (eV)	FWHM (eV)	Assignment
A	399.99	1.64	NR_3_
AuNPs-A	399.72	1.44	NR_3_
	400.95	1.44	NR_3_H^+^
B	399.71	2.00	N ≡ C − R
AuNPs-B	398.70	2.06	N ≡ C − R(δ^−^)
	399.70	2.06	N ≡ C − R

## Supplementary Materials

The following are available online at https://www.mdpi.com/2079-4991/9/5/772/s1, Figure S1: Uv–Vis spectra and calibration curves for complex A (green) and complex B (blue); Figure S2: SEM-EDX analysis on AuNPs-A; Figure S3: ATR data of conjugates systems: (a) AuNPs-A (PTA); (b) AuNPs-B (PCN); Table 1. C1s and P2p spectra data analysis BE, FWHM values and assignments for pristine Cu(I) complexes and AuNPs carriers; Figure S4: XPS P2p spectra confirming the molecular structure stability of A and B complexes; Figure S5: (a) XPS Au4f spectrum of AuNP-A; (b) Cu2p spectra of complex A and AuNP-A (rough data, confirming the stability of Cu(I) complex).
