# Supplementary material for: Highly Hydrophilic Gold Nanoparticles as Carrier for Anticancer Copper(I) Complexes: Loading and Release Studies for Biomedical Applications
Source: Nanomaterials (Basel). 2019 May 20;9(5):772. doi: 10.3390/nano9050772 (PMC6567210; doi:10.3390/nano9050772)

Article

Highly Hydrophilic Gold Nanoparticles as Carrier for Anticancer Copper(I) Complexes: Loading and Release Studies for Biomedical Applications

Ilaria Fratoddi [1], Iole Venditti [2]*, Chiara Battocchio [2], Laura Carlini [2], Simone Amatori [1], Marina Porchia [3]*, Francesco Tisato [3], Federica Bondino [4], Elena Magnano [4], Maura Pellei [5] and Carlo Santini [5]

Figure S1: Uv-vis spectra of (**a**) complex **A** and (**b**) complex **B**; Calibration curves for complex **A** (**c**) and complex **B** (**d**). (The error bars are inserted but not appreciable).

(a)

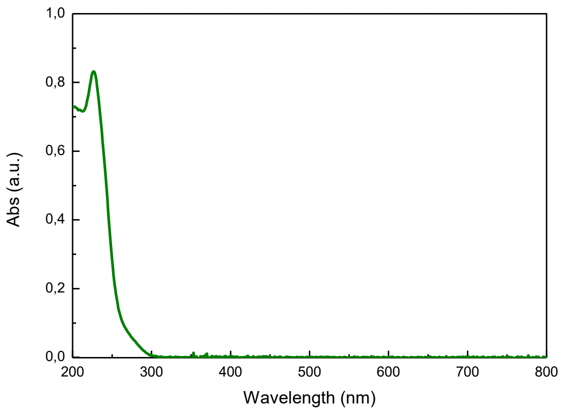

(b)

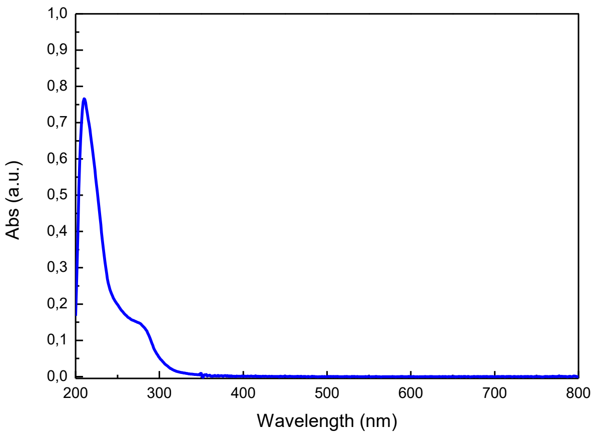

(c)

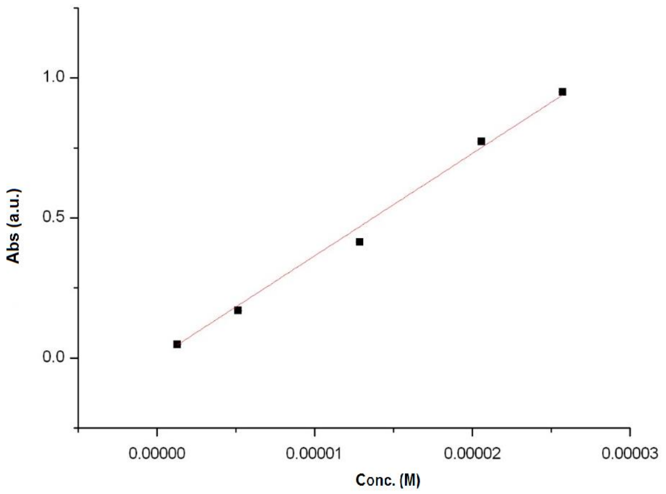
1.0
0.5
0.0
Abs (a.u.)
0.00000
0.00001
0.00002
0.00003
Conc. (M)

(d)

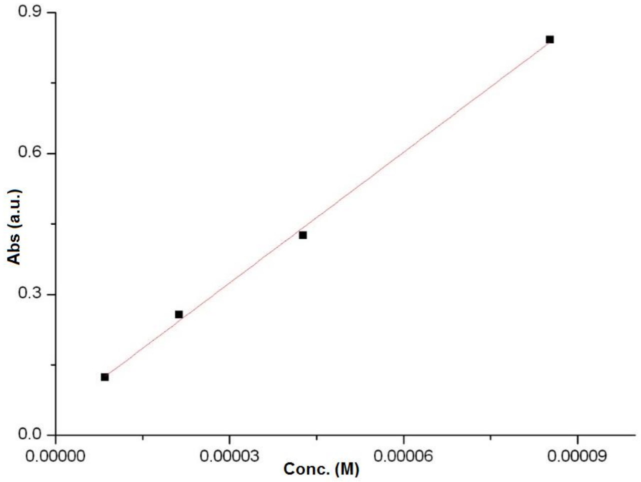
0.9
0.6
0.3
0.0
Abs (a.u.)
0.00000
0.00003
0.00006
0.00009
Conc. (M)

Figure S2: SEM-EDX analysis of AuNPs-**A**

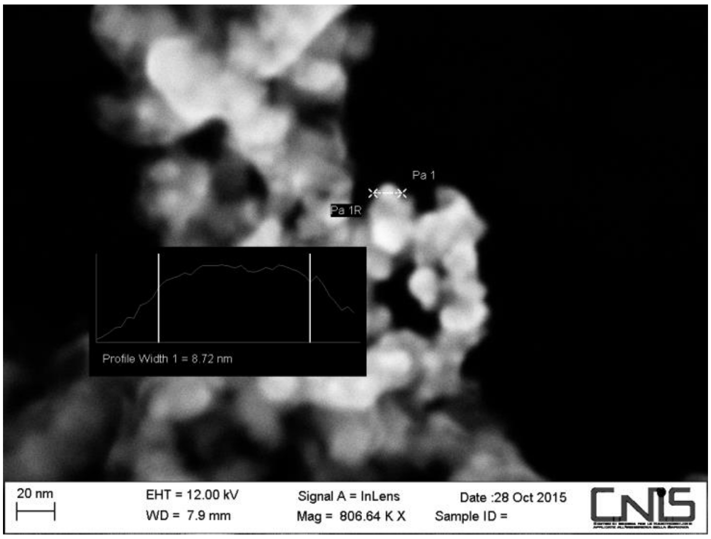

Elemental Microanalysis of AuNPs-**A**

Spectrum	C	Au	Cu	Tot
AuNPs-A	18.5	79.30	2.20	100.00

All result in weight %.

Figure S3: ATR data of conjugates systems: a) ATR spectra of complex **A** (red line) and AuNPs loaded with complex **A** (blue line); b) ATR spectra of complex **B** (pink line) and AuNPs loaded with complex **B** (green line).

a)

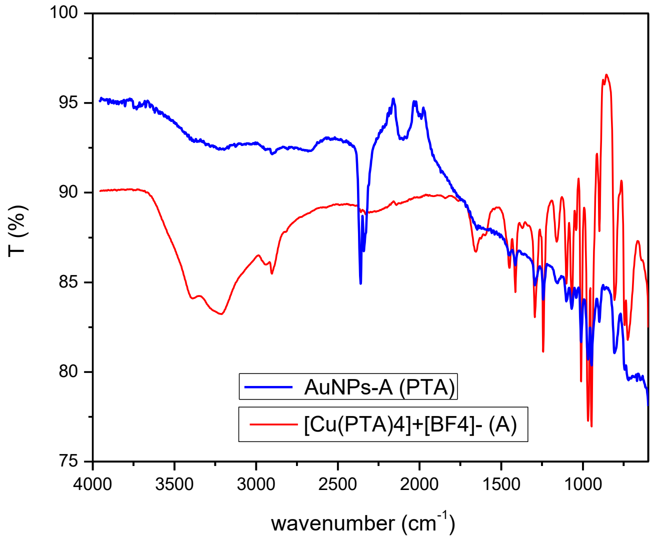

b)

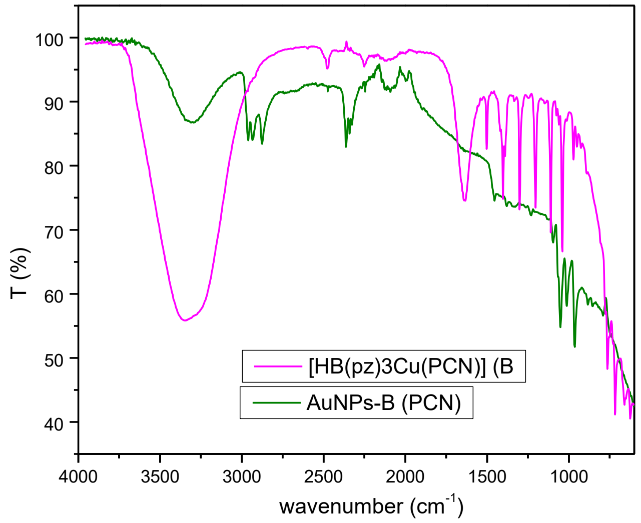

Table S1. C1s and P2p spectra data analysis BE, FWHM values and assignments for pristine Cu(I) complexes and AuNPs carriers

Table S1. C1s and P2p BE, FWHM values and assignments for pristine Cu(I) complexes and AuNPs carriers.

Sample	BE (eV)	FWHM (eV)	Assignment
**A**			
C1s	285.50	1.04	C-P
	286.00	1.04	C-N
P2p	130.43	2.79	P in organic compounds, not oxidized
AuNP-**A**			
C1s	285.00	0.59	C-C
	205.50	0.59	C-P
	286.00	0.59	C-N
P2p	131.11	1.71	P in organic compounds, not oxidized
**B**			
C1s	285.00	1.04	C-C
	286.65	1.04	C-P, C-N
	288.46	1.04	PCN
	290.94	1.04	COO^- impurities
P2p	131.81	1.77	P in organic compounds, not oxidized
AuNP-**B**			
C1s	285.00	1.04	C-C
	286.59	1.04	C-P, C-N
	288.02	1.04	PCN
	288.98	1.04	COO^- impurities
P2p	131.86	1.72	P in organic compounds, not oxidized

Figure S4: XPS P2p spectra, confirming the molecular structure stability of **A** and **B** complexes.

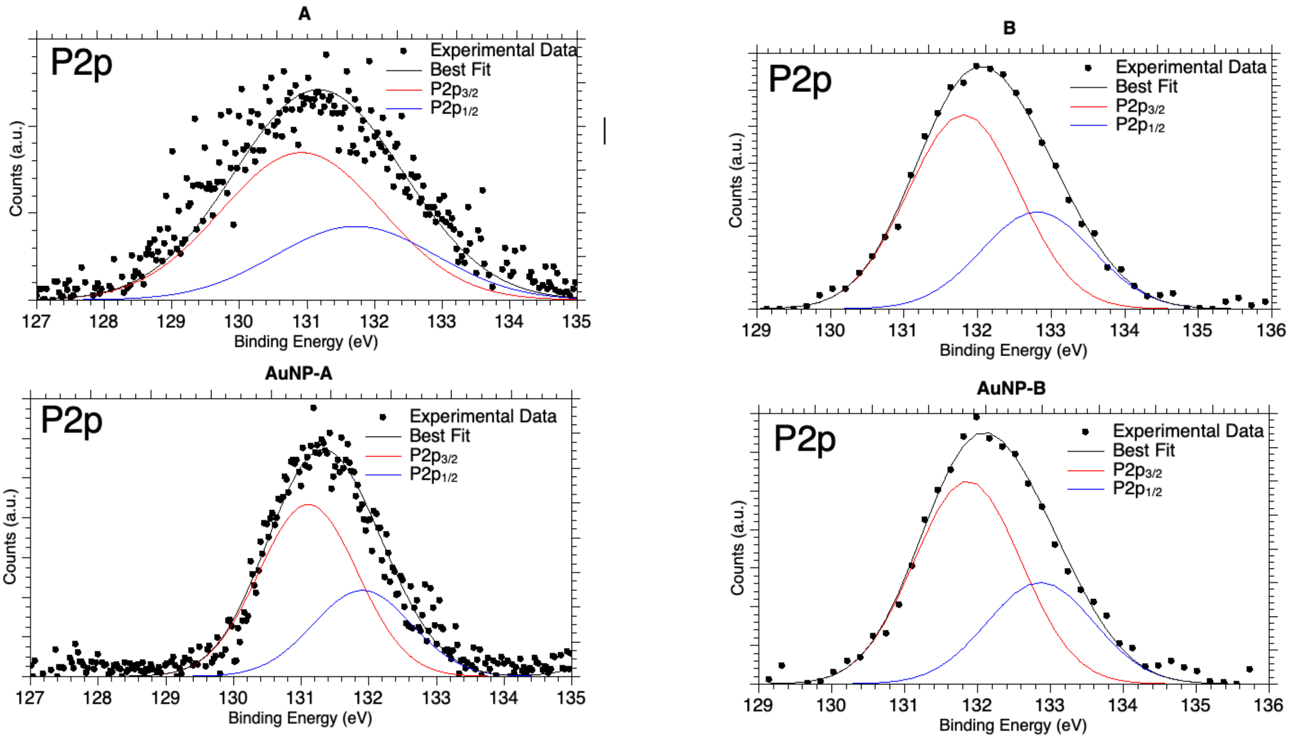

Figure S5: a) XPS Au4f spectrum of AuNP-A. b) Cu2p spectra of complex **A** and AuNP-**A** (rough data, confirming the stability of Cu(I) complex).

a)

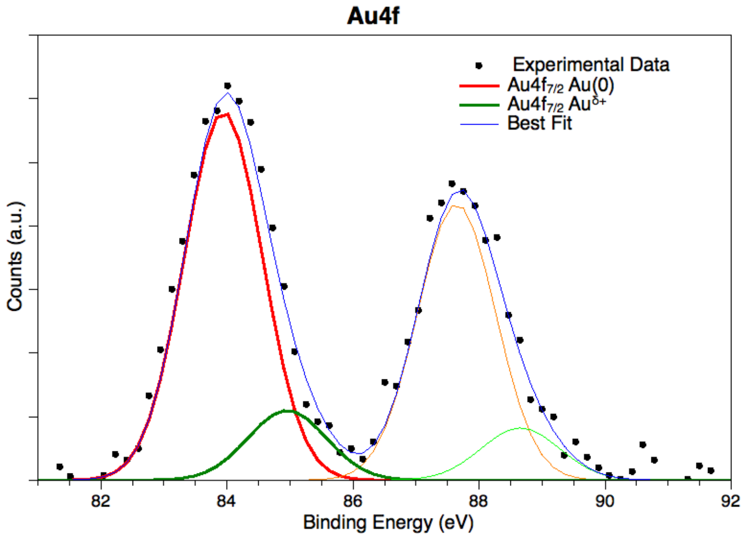

b)

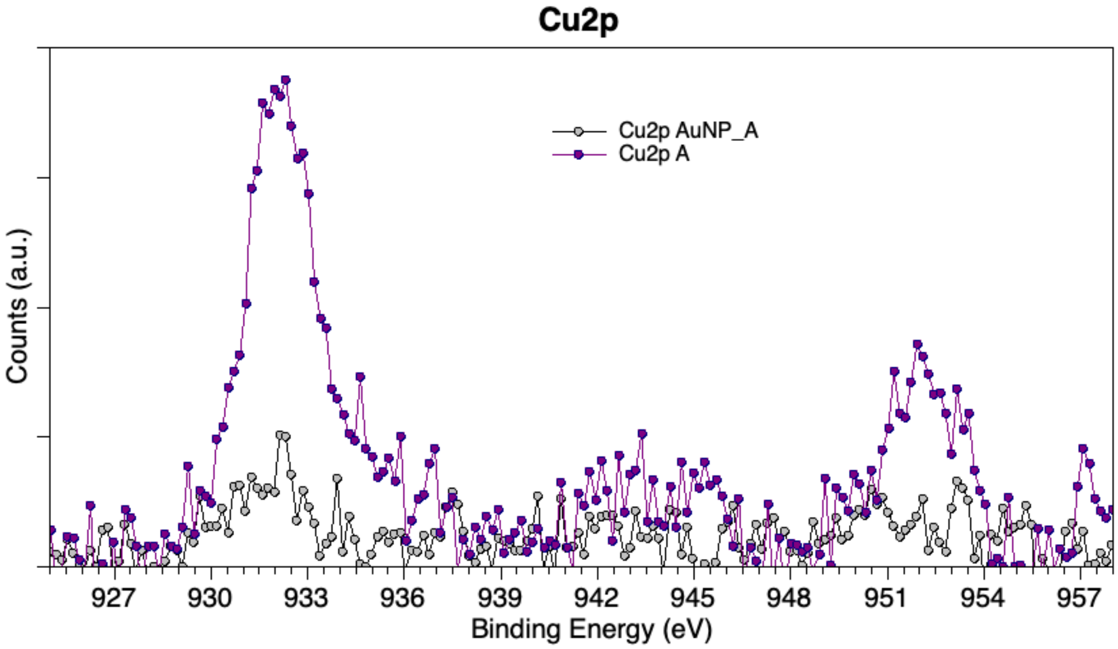

Supplement: Supplementary file 1 [file nanomaterials-09-00772-s001.pdf]
